# Peripheral Blood DNA Methylation Changes in Response to *Centella asiatica* Treatment in Aged Mice

**DOI:** 10.3390/biology14010052

**Published:** 2025-01-10

**Authors:** Olivia Monestime, Brett A. Davis, Cora Layman, Kandace J. Wheeler, Wyatt Hack, Jonathan A. Zweig, Amala Soumyanath, Lucia Carbone, Nora E. Gray

**Affiliations:** 1 BENFRA Botanical Dietary Supplements Research Center, Oregon Health & Science University, Portland, OR 97239, USAdavibr@ohsu.edu (B.A.D.); soumyana@ohsu.edu (A.S.);; 2 Department of Neurology, Oregon Health & Science University, Portland, OR 97239, USA; 3 Department of Medicine, Knight Cardiovascular Institute (KCVI), Oregon Health and Science University, Portland, OR 97239, USAwheelkan@ohsu.edu (K.J.W.); 4 Department of Molecular and Medical Genetics, Oregon Health and Science University, Portland, OR 97239, USA; 5 Division of Genetics, Oregon National Primate Research Center, Beaverton, OR 97006, USA

**Keywords:** aging, DNA methylation, *Centella asiatica*, epigenetics

## Abstract

Alterations in epigenetic modifications, like DNA methylation, in peripheral blood could serve as a useful, minimally invasive biomarker of the effects of anti-aging interventions. This study explores this potential with a water extract of the botanical *Centella asiatica* (CAW). Eighteen-month-old mice were treated with CAW in their drinking water for 5 weeks alongside vehicle-treated eighteen-month-old C57BL6 mice. Reduced representation bisulfite sequencing (RRBS) was used to identify genome-wide differential methylation in the blood of CAW-treated aged mice compared to vehicle-treated aged mice. Our results showed a distinct enrichment of differentially methylated regions (DMRs) nearby genes involved in biological processes relevant to aging (i.e., antioxidant response, metabolic regulation, cellular metabolism). A distinct difference was observed between males and females in both the number of methylation sites and the state of methylation. Moreover, genes nearby or overlapping DMRs were found to be enriched for biological processes related to previously described cellular effects of CAW in the mouse brain (i.e., antioxidant response, metabolic regulation, calcium regulation, and circadian rhythm). Together, our data suggest that the peripheral blood methylation signature of CAW in the blood could be a useful, and readily accessible, biomarker of CAW’s effects in aging.

## Simple Summary

We investigated the DNA methylation signature in the peripheral blood of aged mice treated with the cognitive-enhancing water extract of *Centella asiatica (CAW)*. CAW-treated mice showed distinct methylation and enrichment in, and nearby, genes associated with increased lifespan and processes associated with healthy aging including cellular and metabolic regulation, DNA repair, and energy homeostasis. Many of these pathways are consistent with previously reported effects of *Centella asiatica* in the brain suggesting that epigenetic changes in peripheral blood may be a useful biomarker to use in future studies.

## 1. Introduction

Aging remains the greatest risk factor for developing many somatic and neurological diseases due to the degradation of critical immunosuppressive, neurological, and metabolic pathways related to longevity [1,2]. As the aging population continues to grow worldwide, so too has the search for interventions that can promote resilience to age-related changes. *Centella asiatica* (L.) urban is an Ayurvedic herb that originates from Southeast Asia and has been used for centuries in the treatment of a variety of ailments related to neuronal health and mitigating age-related cognitive decline [3,4]. Previous work in our lab has shown that a water extract of *Centella asiatica* (CAW) administered in the drinking water of mice significantly alleviates age-related behavioral alterations, including improving cognitive performance and decreasing anxiety [5,6,7]. To continue developing CAW for eventual therapeutic use, it is essential to identify translationally relevant, minimally invasive biomarkers to track the effects of the extract during aging.

Biological markers of aging are reflected in epigenetic modification to DNA. Epigenetic changes are chemical modifications that induce changes in gene expression, without directly altering DNA sequence [8]. Age-associated epigenetic modifications in the genome have been used to build epigenetic clocks that have been proven to be able to reliably measure biological aging [9,10] and have been validated as useful biomarkers of aging in humans as well as rodents [9,11,12,13,14,15,16].

DNA methylation changes are also associated with age-related alterations in brain function, including cognitive decline [12,17]. Studies in both humans and rodents have linked methylation changes in brain tissues to impairments in learning, memory, and neuronal health with advancing age [13,14,17]. A study from our own group showed that some DNA methylation patterns evident in the brains of aging mice are mirrored in the blood as well [18].

Considering these findings, as well as the reported effects of CAW on age-related brain function [5,6,7], this study sought to determine the effects of CAW on changes in DNA methylation in peripheral blood to identify a signature of response to the extract in aged C57BL6 mice.

## 2. Materials and Methods

### 2.1. CAW

CAW was prepared and administered to the mice as described in our previous publication [5]. Briefly, 4 kg of *Centella asiatica* (dried aerial parts) was acquired from Oregon’s Wild Harvest (Redmond, OR, USA) and boiled for 90 min in 50 L deionized water. The liquid was filtered using a filter bag (McMaster-Carr #5162K112 filter bag) to remove insoluble materials and debris. The filtrate was frozen in aluminum baking trays and lyophilized in batches to yield a total of 820 g of CAW. Voucher samples of the starting plant material are stored at the Oregon State University Herbarium (voucher number OSC-V265416) and in our laboratory at Oregon Health & Science University (voucher number BEN-CA-6), as are voucher samples of the dried CAW extract batches (voucher numbers BEN-CAW 7, 8, and 9).

### 2.2. Mice

Eighteen-month-old male and female C57BL6 mice were obtained from the National Institute of Aging’s Aged Rodent Colony. The sample size was determined based on previous work by our group where we detected significant age-related DNA methylation changes with 6 mice in each age group [18]. Because this study was focused on treatment effects following CAW exposure, this number was doubled to increase the chances of detecting group differences. Mice were maintained in a climate-controlled facility with a 12 h light/dark cycle and water and food (AIN-93M; Dyets Inc., Bethelem, PA, USA) were supplied ad libitum. All methods were performed in correspondence with the NIH guidelines for the Care and Use of Laboratory Animals and were approved by the Institutional Animal Care and Use Committee of the Portland VA Medical Center.

Mice were randomly assigned to be treated with either 0 or 10 mg/mL CAW in their drinking water (supplied ad libitum) for a total of 5 weeks (*n* = 12 of each sex per group). Drinking water containing CAW was replaced twice weekly, i.e., every 3 or 4 days. At the conclusion of treatment, whole blood was collected via cardiac puncture and placed in EDTA-treated tubes.

### 2.3. DNA Extraction and Quality Validation

DNA was extracted by Omega Biosciences using Mag-Bind Blood & Tissue DNA HDQ 96 Kit (Omegabiotech) and quality was confirmed by Nanodrop and Picogreen. The DNA samples were transferred to Knight Cardiovascular Institute (KCVI) epigenetics core and stored at −80 °C until analysis. DNA samples were measured for quality and concentration on the Qubit Fluorometer (ThermoFisher, Waltham, MA, USA) and Nanodrop (ThermoFisher). Researchers were blinded to the group allocations and all samples were of sufficient quality so that none were excluded from analysis.

### 2.4. Reduced Representation Bisulfite Sequencing (RRBS) Library Generation

To analyze DNA methylation, we used reduced representation bisulfite sequencing (RRBS) [19], a highly cost-effective, genome-wide approach that allows us to capture key regulatory regions, including promoters, CpG islands, and CpG island shores. RRBS libraries were generated by the KCVI Epigenetics Core at Oregon Health and Science University (OHSU) using established methods [18,20]. The samples were processed in a 48-well plate format, using 100 ng of DNA as input for each reaction. Digestion with *MSPI* restriction enzyme (New England Biolabs [NEB]) was carried out for 2 h, resulting in sticky ends starting with CpG. The fragments went through end repair and a-tailing, adaptor ligation, and U-excision using the NEBNext Ultra II Modules and NEBNext Methylated Adaptor for Illumina. The libraries were size-selected to approximately 300 bp using the Ampure XP magnetic beads (Beckman Coulter, Brea, CA, USA) and then went through bisulfite conversion using the EZ-96 DNA Methylation Gold Kit (Zymo Research, Irvine, CA, USA). The bisulfite-converted DNA was then PCR amplified using the Q5U master mix and Multiplex Oligos for Illumina from NEB, and the PCR reaction was cleaned using the Ampure XP magnetic beads (Beckman Coulter). The libraries were submitted to the Massively Parallel Sequencing Shared Resource (MPSSR) at OHSU for paired-end sequencing on the Illumina NextSeq 500 platform to obtain roughly 60 million reads per library.

### 2.5. Differential Methylation Analysis

Sequencing reads were analyzed as described in Carbone et al., 2019 [20]. Briefly, after evaluation with FastQC [21], reads were trimmed with TrimGalore specifying the -rrbs parameter. Trimmed reads were aligned to the mouse reference genome from Ensembl GRCm38 with Bismark [22], which also performs methylation calling on every covered cytosine.

MethylKit [23] was used to perform differential methylation analysis. Cystosines were filtered to remove those with less than 10× coverage or more than the 99.9th percentile of highest coverage. The genome was then tiled into 1 kb non-overlapping regions, and the methylation information (using covered CpGs) was averaged over each region. Also, each region was required to have a minimum of 5 covered CpGs. The remaining regions were required to be shared in at least 8 samples per group to be retained for DMR analysis, resulting in 95,406 regions. DMRs of interest were selected based on q-value (<0.1) and percent methylation difference (>10%) cutoffs. DMRs were annotated to locations in nearby genes using ChIPseeker [24] and the Ensembl GRCm38 annotation gtf file. We defined promoters as 3 kb upstream from a transcription start site (TSS). If the DMR overlapped an annotated promoter, exon, or intron, then it was considered as overlapping that given gene feature. In some instances, a DMR can overlap more than one feature; for example, a DMR that spanned the promoter region, and the first exon of a gene would be considered as overlapping both features. For intergenic DMRs, the closest gene and the distance between the DMR and TSS were also annotated.

### 2.6. Characterization of DMRs

In order to identify pathways enriched in DMRs, we used the publicly available tool EnrichR (https://maayanlab.cloud/Enrichr/ (accessed on 9 September 2023) with Gene Ontology (GO) Biological Process 2023, which allows for a comprehensive, computational, model of gene functions across all organisms [25]. We also examined potential methylation changes in transcription factor binding motifs that could be involved in the misregulation of key genes/pathways, using HOMER (Hypergeometric Optimization of Motif Enrichment) version 4.11 [26]. Enriched motifs with a Benjamini value of less than 0.05 were identified.

## 3. Results

### 3.1. CAW Treatment Results in Variable Numbers of Differentially Methylated Regions in the Peripheral Blood of Aged Male and Female Mice

**Figure 1:**
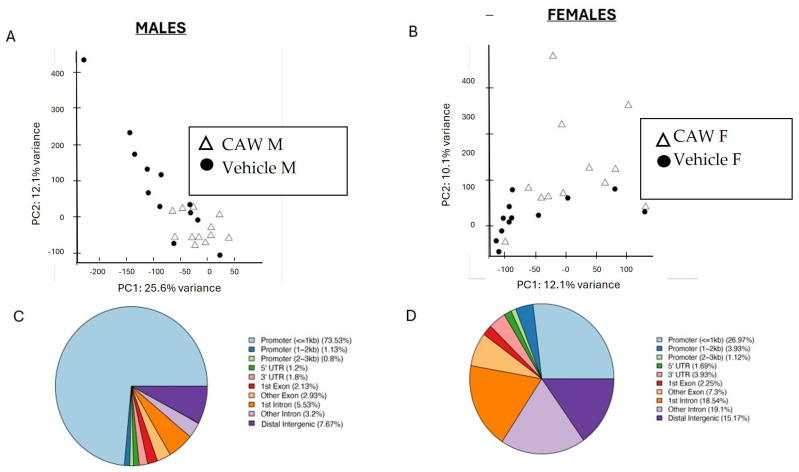
Principal Component Analysis of CpG methylation sites in aged male (**A**) and female (**B**) mice, grouped by treatment. Distribution of differentially methylated regions (DMRs) along CpG sites across the genome in males (**C**) and females (**D**).

To explore the methylation signature associated with CAW administration in aged mice, we generated RRBS data from the peripheral blood of aged mice (18 months) treated with CAW and their vehicle-treated littermates. When looking at global DNA methylation, we observed a good separation between CAW- and vehicle-treated groups of each sex, as determined by Principal Component Analysis (Figure 1A,B).

We first identified differentially methylated regions (DMRs) using q-value < 0.1 and percent methylation difference > 10%. We first found that there were many more (almost 10-fold) DMRs in male CAW mice compared to female CAW mice (1500 vs. 178, respectively), with most of the DMRs (1489, 99%) being hypomethylated in CAW treatment males, whereas most DMRs (107, 60%) were hypermethylated in response to CAW in females (Table 1). In both males and females, the majority of DMRs were found in promoter regions, with this enrichment being more pronounced in males (Figure 1C). Intronic and distal intergenic regions were enriched within the DMRs of female CAW mice (Figure 1D).

### 3.2. DMRs Are Enriched by CAW in Distinct Pathways in Male and Female Aged Mice

In order to determine if DMRs were enriched for specific biological processes in response to CAW treatment, we used EnrichR and Gene Ontology (GO) Biological Processes [25]. The top 15 enriched pathways for DMRs occurring anywhere in the gene are shown in Table 2 for each sex. In male CAW mice, the most significantly enriched pathways were related to transcriptional regulation, i.e., “Regulation of DNA-templated Transcription”, “Regulation of Transcription by RNA Polymerase II”, and “Nucleic Acid-Templated Transcription”. In contrast, in females, the most significantly enriched pathways were associated with cellular responses, particularly those related to oxidative stress regulation, growth hormone signaling, and cytoskeletal organization, with “Cellular Response to Oxygen-Containing Compound” at the top of the list.

Notably, several of the top 15 enriched biological processes and pathways in both male and female CAW mice have been previously reported as potential mechanisms of *Centella asiatica* (indicated in bold text in Table 2) [6,7,27,28,29,30,31,32,33,34]. When focusing on the “Cellular Response to Oxidative Stress” pathway in male CAW mice, we observed that the genes from this pathway are tightly associated with the reported effects of *Centella asiatica* (Table 3).

Genes in enriched pathways include several antioxidant enzymes, as well as ones involved in mitochondrial function, metabolism, and energy homeostasis, along with genes associated with calcium homeostasis and circadian rhythm (Table 3)—all of which have been reported previously to be affected biologically by *Centella asiatica* [31,35,36,37,38,39,40,41,42]. All the genes in this pathway were hypomethylated in CAW mice (Table 3). Hypomethylation is typically associated with increased gene expression, particularly if the hypomethylation occurs in the promoter, but transcription can also be impacted by methylation changes in other gene regions [15,43].

Moreover, the “Cellular Response to Oxygen Containing” pathway in female CAW mice (Table 4) includes genes associated with reported antioxidative effects of CAW in the literature [3,4,6,27,28,30,32,44]. Specifically, we found that *Il10*, the anti-inflammatory cytokine, was hypomethylated, while genes associated with a pro-inflammatory response were hypermethylated. Genes related to vascular function and calcium signaling were also differentially methylated in response to CAW.

A list of all genes associated with the top 15 GO Biological Processes that have been reported to be affected by *Centella asiatica* in the literature can be found in Supplementary Materials Tables S1 and S2.

### 3.3. DMRs Are Enriched in Different Transcription Factor Binding Motifs in CAW-Treated Male vs. Female Mice

Genomics regions overlapping with DMRs might represent regulatory regions that might be available for binding by specific transcription factors and their methylation status might impact the binding status by making chromatin more or less accessible [45]. We therefore investigated the possible enrichment of specific transcription factor binding motifs in our DMRs using the software HOMER [26]. In both male and female CAW mice, HOMER analysis revealed enriched transcription factor binding site motifs, and the top 15 are shown in Table 5.

In CAW mice, several of the top motifs in hypermethylated DMRs belonged to the Specialty Protein (SP) and Kruppel-like Factors (KLF) families of transcription factors, while, in female CAW mice, many belong to the E-twenty-six (ETS) or Interferon Regulatory Factor (IRF) families, which are transcription factors families involved extensively in immunoregulatory and metabolic functions (Table 5) [46,47,48].

Only two transcription factor binding motifs were enriched in DMRs that were hypomethylated in male CAW mice, whereas, in females, many more were enriched in DMRs that were hypomethylated by CAW, and, again, many of these were associated with the ETS and IRF families of transcription factors (Table 5).

## 4. Discussion

We have recently shown that *Centella asiatica* (CAW) is able to elicit robust improvements in learning, memory, and executive function in aged mice [5,6,7]; as such, this botanical represents an enticing therapeutic candidate to battle cognitive decline occurring with aging. As we continue to develop CAW for clinical applications, it is critical to identify translationally relevant biomarkers to track its effects. To that end, here, we seek to determine the DNA methylation signature of CAW treatment in the peripheral blood of aged mice. Prior work from our group has shown that male and female mice can exhibit differential magnitudes of behavioral changes and alterations in protein and gene expression in response to CAW [5,27,49,50]. We have reported that CAW improves anxiety in aged female mice but not in aged male mice, despite improving executive function and memory in both sexes [5] and, in the mouse model of beta-amyloid accumulation, the magnitude of memory improvement, as well as changes in synaptic and antioxidant gene expression, induced by CAW has been shown to vary between the sexes [27,50]. Likewise, analysis of the metabolomic pathways that were altered by CAW in the brain of beta-amyloid-expressing mice also revealed sex differences [49]. For this reason, we decided to analyze DNA methylation changes in each sex separately. As anticipated, this epigenetic signature of CAW treatment was not identical in male and female mice. Overall, male CAW mice exhibited significantly more DMRs across the genome than female mice and they were associated mainly with gene GO Biological Processes related to transcription, calcium homeostasis, and response to oxidative stress. DMRs in female CAW mice were associated with response to oxygen-containing compounds but also with various signal transduction pathways and protein transport.

Although it was not the focus of this study, age-related sex differences were also observed between young and aged vehicle-treated mice (see the data in Supplementary Materials). The number and distribution of DMRs varied between the aged male and female animals (Supplementary Materials Table S3 and Figure S1). The GO Biological Processes that were enriched for those DMRs were also distinct in male and female aged animals (Supplementary Materials Table S4), as were the transcription factor binding motifs found within those regions (Supplementary Materials Tables S5 and S6).

Despite these differences between the sexes, for both sexes, there were DMRs in genes associated with mechanistic pathways that were reported to be affected by *Centella asiatica* in the brain [6,7,12,27,28,29,30,31,33,34,48,49,50,51]. This supports the idea that peripheral blood DNA methylation might reflect changes that occur in the brain and could be useful as a clinical metric to monitor in human studies as CAW continues to be developed for therapeutic use. In male CAW mice, many genes within the biological process of “Cellular Response to Oxidative Stress” were, unsurprisingly, involved in antioxidant response. These included enzymes in glutathione homeostasis, which were all hypomethylated in the CAW-treated group. Because hypomethylation is usually associated with increased gene expression, this is consistent with the effects of CAW on the expression of antioxidant enzymes in the brain reported by our group, as well as reports from other groups on the effects of other *Centella asiatica* extracts on brain levels of glutathione and antioxidant enzymes in rodents [6,7,27,34,35,52,53]. In that same “Cellular Response to Oxidative Stress” process, several genes involved in mitochondrial function and energy homeostasis were also hypomethylated in the blood of CAW mice. This is likewise consistent with reports from our group and others of the effects of CAW and other *Centella asiatica* extracts on mitochondrial endpoints in the brains of treated rodents. In summary, multiple DNA methylation changes apparent in the blood may reflect parallel alterations in the same mechanistic pathways in brain tissue; however, future experiments are needed to definitely confirm this link between methylation patterns in the blood and the expression of the same gene in the brain of CAW-treated animals.

Similar parallels between blood methylation patterns and mechanistic pathways known to be altered by *Centella asiatica* in the brain were also observed in female CAW mice. In the biological process, “Cellular Response to Oxygen-Containing Compounds”, several genes related to inflammation were differentially methylated in the CAW-treated mice. *Wnt5b*, *Cd80*, and *Plcg2* are all associated with promoting inflammation and were all hypermethylated in the CAW mice, while *Il10*, an anti-inflammatory cytokine, was hypomethylated. This mirrors prior reports of the anti-inflammatory effects of *Centella asiatica* in the brain and brain cells [36,37,38,52,53].

To explore the possible function of the DMRs identified in this study, we investigated the possibility that these regions were enriched in transcription factor binding sites [15]. Even in this case, we identified a difference between the two sexes.

In female CAW mice, several of the transcription factor binding motifs that were significantly enriched within DMRs belonged to the ETS and IRF families. ETS transcription factors have been shown to be involved in a variety of cellular processes of inflammation and cellular stress response, with increased activation linked to age-related inflammatory pathways and neurodegeneration [54]. They have also been implicated in aging, as several factors within the family have been shown to heavily influence longevity-promoting pathways in *Drosophila* and *C. elegans* [46,55]. Lastly, the link to aging is also evident in humans, where expression of *ETS1* was found to be associated with metabolic energy saving and healthy aging in long-lived individuals [47]. IRF transcription factors are crucial regulators of immune responses and have shown age-associated dysregulation in neuroinflammatory contexts, contributing to increased risk of neurodegenerative disease [48,56]. IRF4 and 5 are known to regulate the activation of macrophages and are involved in cerebral inflammatory responses, and their relative abundance in the brain is altered with age in mice [57]. Other studies using rodent models have also shown that reductions in IRF expression contribute to weakened defense mechanisms against oxidative stress, which is heavily implicated as a driver in age-related decline in brain health [48].

In male CAW mice, many binding motifs for the KLF (Kruppel-like Factors) transcription factor family were found to be enriched in our DMRs. KLFs belong to a diverse group of transcription factors involved in various biological processes, including genomic integrity, epigenetic and cellular reprogramming, mitochondrial health, microglial activation, and in turn systemic inflammation—all of which are molecular mechanisms involved in aging [58]. In recent years, there has been growing evidence for the role of KLFs in modulating the fundamental progression of aging, particularly regarding vascular aging [58]. KLF14 has been linked to aging with its expression decreasing with age [59]. Additionally in a senescence-accelerated mouse line, KLF14 protein in the brain was decreased, whereas in a senescence-resistant mouse line, its expression was increased [60]. Moreover, KLF14 knockout mice showed accelerated age-related cognitive decline, hair loss, cardiac hypertrophy, and increased tissue fibrosis and exhibited higher rates of mortality [60]. Conversely, overexpression of KLF14 in in vitro models has been shown to contribute to pro-apoptotic signaling processes [61,62]. KLF3 is another member of the KLF family that has likewise been implicated in the direct regulation of longevity in *C.elegans*, with expression of KLF3 found to be a requirement for normal lifespan, and overexpression related to beneficial longevity effects [63,64]. In our study, both KLF14 and KLF3 transcription factor binding motifs were enriched in hypomethylated DMRs in female CAW mice.

Binding sites for the Specialty Protein (SP) family of transcription factors were also identified in our analysis of male CAW mice. This family has also been shown to be involved in a variety of cellular and metabolic regulatory processes involved in aging [63,65]. SP1 and SP1-like transcription factors share many of the same binding motifs as KLFs, often competing for the same site with opposing regulatory functions [65]. SP transcription factors have been shown to mediate cellular senescence and inflammatory response in nematodes and *Drosophila* [65,66]. SP1 has been shown to be responsible for aging-dependent alterations in nucleocytoplasmic trafficking [51] and it has been reported to be downregulated during cellular senescence [66]. Future studies are needed to determine if changes in SP transcription factor activity may mediate the beneficial effects of CAW.

Although we observe this intriguing enrichment of DMRs in biologically relevant transcription factor binding motifs, our study cannot determine if any of the transcription factors themselves are differentially expressed and/or differentially binding these regions in response to CAW exposure. Future studies will be needed to confirm whether this is true, as well as to establish if methylation changes in correspondence to the identified DMRs might be responsible for the differential regulation of pathways via differential binding of specific transcription factors.

## 5. Conclusions

Taken together, the results from this study provide evidence for a peripheral blood DNA methylation signature in response to CAW. The biological processes identified here are in line with the biological responses yielded by CAW. However, more work is needed to understand how the molecular signatures observed in peripheral blood reflect those in the brain. These future studies could identify links between the DNA methylation changes observed in this study and the behavioral and cellular changes in the brain previously observed by our group in CAW-treated mice. Such studies would provide important support for peripheral blood as a useful biomarker of target engagement to investigate as CAW continues toward clinical development.

## Figures and Tables

**Table 1:** Differentially methylated regions in CAW-treated aged mice compared to vehicle-treated aged mice; DMRs with a q-value (<0.1) and percent methylation difference (>10%) were included in this analysis.

Comparison (CAW vs. Control)	Total Number Significant DMRs	Number of Significant Hypomethylated DMRs	Number of Significant Hypermethylated DMRs
Old Male CAW vs. Control	1500	1489	11
Old Female CAW vs. Control	178	71	107

**Table 2:** Top 15 enriched GO Biological Processes found anywhere in the genome of male and female CAW mice. Bold text and gray fill indicate processes reported to be affected by *Centella asiatica* in the literature.

Male			Female		
Term	*p*-Value	Adjusted *p*-Value	Term	*p*-Value	Adjusted *p*-Value
Regulation Of DNA-templated Transcription (GO:0006355)	<0.001	0.080	**Cellular Response To Oxygen-Containing Compound**	**<0.001**	**0.246**
Regulation Of Transcription By RNA Polymerase II	<0.001	0.093	Growth Hormone Receptor Signaling Pathway Via JAK-STAT	0.002	0.246
Negative Regulation Of Nucleic Acid-Templated Transcription	<0.001	0.102	Cortical Cytoskeleton Organization (GO:0030865)	0.002	0.246
Positive Regulation Of DNA-templated Transcription	<0.001	0.117	Regulation Of Protein Transport (GO:0051223)	0.002	0.246
**Regulation Of Dendritic Spine Development (GO:0060998)**	**<0.001**	**0.243**	Regulation Of Neuron Migration (GO:2001222)	0.002	0.246
**Cellular Response To Oxidative Stress (GO:0034599)**	**<0.001**	**0.243**	Positive Regulation Of Membrane Invagination (GO:1905155)	0.002	0.246
Positive Regulation Of Transcription By RNA Polymerase II	<0.001	0.243	**Regulation Of Dendritic Cell Apoptotic Process (GO:2000668)**	**0.003**	**0.246**
mRNA Splice Site Recognition (GO:0006376)	<0.001	0.275	**Nerve Growth Factor Signaling Pathway (GO:0038180)**	**0.003**	**0.246**
Regulation Of Neuron Differentiation (GO:0045664)	<0.001	0.399	**cAMP Biosynthetic Process (GO:0006171)**	**0.003**	**0.246**
**Calcium-Ion Regulated Exocytosis (GO:0017156)**	**0.002**	**0.490**	Positive Regulation Of Leukocyte Apoptotic Process	0.003	0.246
**Calcium Ion-Regulated Exocytosis Of Neurotransmitter**	**0.002**	**0.490**	Positive Regulation Of Phagocytosis, Engulfment	0.003	0.246
Myeloid Cell Development (GO:0061515)	0.002	0.490	Rap Protein Signal Transduction (GO:0032486)	0.003	0.246
Regulation Of Regulated Secretory Pathway (GO:1903305)	0.002	0.490	Regulation Of Phagocytosis, Engulfment (GO:0060099)	0.003	0.246
**Positive Regulation Of Dendritic Spine Development**	**0.002**	**0.490**	Cellular Response To Forskolin (GO:1904322)	0.003	0.246
Forebrain Neuron Differentiation (GO:0021879)	0.003	0.490	Response To Forskolin (GO:1904321)	0.003	0.246

**Table 3:** Genes containing significant DMRs in “Cellular Response to Oxidative Stress” in male CAW mice. Bold text and gray fill indicate association with a reported effect of *Centella asiatica* in the literature.

		Meth Diff	*p* Value	q Value	Location
	*Pdgfra*	−12.225	<0.001	0.001	Promoter (<=1kb)
***Antioxidant***	***Gpx1***	**−18.350**	**<0.001**	**0.005**	**Promoter (<=1kb)**
	***Gpx7***	**−10.380**	**0.002**	**0.014**	**Promoter (<=1kb)**
	***Gsr***	**−12.527**	**0.001**	**0.011**	**Exon**
	*Pxn*	−11.239	0.032	0.100	Distal Intergenic
	*Dapk1*	−10.365	<0.001	0.001	Promoter (<=1kb)
***Mitochondrial***	***Pycr2***	**−13.480**	**0.007**	**0.036**	**Distal Intergenic**
	*Pex10*	−13.653	<0.001	0.002	Promoter (<=1kb)
	*Pex5*	−13.208	<0.001	0.003	Promoter (<=1kb)
***Energy metabolism, angiogenesis, vascularization***	***Hif1a***	**−14.968**	**<0.001**	**0.006**	**Promoter (<=1kb)**
	*Mmp9*	−10.890	<0.001	0.003	Promoter (1-2kb)
	*Zfp36l2*	−11.104	0.001	0.009	Promoter (<=1kb)
***Calcium signaling***	***Camkk2***	**−16.105**	**<0.001**	**0.003**	**Intron**
***Circadian rhythm***	***Setx***	**−14.958**	**0.002**	**0.015**	**Promoter (<=1kb)**
	*Rad52*	−26.128	<0.001	<0.001	Promoter (<=1kb)
	*Zc3h12a*	−16.137	0.002	0.015	Distal Intergenic
	*Mgat3*	−10.921	0.010	0.046	Intron
***Energy homeostasis***	***Sqstm1***	**−19.144**	**<0.001**	**<0.001**	**Promoter (<=1kb)**
	*Atf4*	−13.091	<0.001	0.001	Promoter (<=1kb)

**Table 4:** Genes containing significant DMRs in “Cellular Response to Oxygen-Containing Compounds” pathway in CAW female mice. Bold text and gray fill indicate association with a reported effect of *Centella asiatica* in the literature.

		Meth Diff	*p* Value	q Value	Location
**Anti-inflammatory**	**Il10**	**−13.194**	**0.007**	**0.095**	**Distal Intergenic**
**Pro-inflammatory**	**Wnt5b**	**10.588**	**0.001**	**0.051**	intron
	**Cd80**	**10.874**	**<0.001**	**0.028**	intron
	**Plcg2**	**10.858**	**0.002**	**0.060**	**Distal Intergenic**
	Rapgef1	−23.373	0.002	0.063	intron
	Rapgef2	10.176	0.003	0.065	Promoter (<=1kb)
**Vascular endothelial and smooth muscle interactions**	**Iqgap1**	**−11.723**	**0.005**	**0.083**	**Intron**
	Lcp1	−12.265	0.001	0.047	Promoter (<=1kb)
**Calcium signaling related**	**Adcy7**	**13.319**	**<0.001**	**0.008**	**intron**
	**Ryr3**	**10.314**	**0.001**	**0.052**	**Promoter(<=1kb)**
	**Camkk2**	**11.250**	**0.001**	**0.047**	**intron**

**Table 5:** Enriched mammalian transcription factor binding motifs in hypomethylated genes (red text) and hypermethylated genes (blue text) in the blood of CAW mice.

Males							Females						
Transcription Factor Name		*p* Value	q Value	% of Target Sequences	% of Background Sequences	Fold Over Background	Transcription Factor Name		*p* Value	q Value	% of Target Sequences	% of Background Sequences	Fold Over Background
Sp5	*Specialty Protein*	<0.001	<0.001	69.91%	57.55%	1.21	Elk4(ETS)	*E twenty six*	<0.001	< 0.001	42.86%	24.00%	1.79
Sp2	*Specialty Protein*	<0.001	<0.001	81.13%	70.90%	1.14	NF1(CTF)	*CCAAT box-binding transcription factor*	<0.001	< 0.001	33.33%	17.25%	1.93
Sp1	*Specialty Protein*	<0.001	<0.001	35.39%	24.89%	1.42	ETV4(ETS)	*E twenty six*	<0.001	< 0.001	64.29%	46.45%	1.38
KLF1	*Kruppel-like factors*	<0.001	<0.001	63.67%	52.49%	1.21	Elk1(ETS)	*E twenty six*	<0.001	< 0.001	41.07%	25.01%	1.64
DPL-1	*Doppel Protein(s)*	<0.001	<0.001	54.40%	43.53%	1.25	AT5G05550(Trihelix)	*activating transcription factor*	<0.001	0.001	61.90%	44.91%	1.38
AT5G23930	*Activating transcription factor*	<0.001	<0.001	77.23%	67.44%	1.15	Fli1(ETS)	*E twenty six*	<0.001	0.007	60.12%	44.67%	1.35
ATAF1	*Activating transcription factor*	<0.001	<0.001	78.51%	69.32%	1.13	Unknown3	*Unknown*	<0.001	0.007	14.29%	5.82%	2.46
AT2G15740	*Activating transcription factor*	<0.001	<0.001	30.36%	21.69%	1.40	ERG(ETS)	*E twenty six*	0.001	0.019	72.02%	58.28%	1.24
E2F4	E2F Family	<0.001	<0.001	37.34%	28.25%	1.32	IRF8(IRF)	*Interferon Regulatory Family*	0.001	0.036	22.62%	12.82%	1.76
Maz(Zf)	*Zinc-finger protein*	<0.001	<0.001	77.10%	68.28%	1.13	At4g18890(BZR)	*Brassinazole-resistant*	0.001	0.056	14.29%	6.89%	2.07
KLF3	*Kruppel-like factors*	<0.001	<0.001	44.12%	34.80%	1.27	IRF1(IRF)	*Interferon Regulatory Family*	0.001	0.068	11.90%	5.37%	2.22
KLF14	*Kruppel-like factors*	<0.001	<0.001	84.82%	77.15%	1.10	IRF2(IRF)	*Interferon Regulatory Family*	0.01	0.12	9.52%	4.05%	2.35
CAMTA	*Calmodulin binding transcription activator*	<0.001	<0.001	31.70%	23.43%	1.35	Mef2c(MADS)	*Minichromosome maintenance 1, agamous, deficiens, serum response factor*	0.01	0.131	22.62%	13.99%	1.62
MYB88	*Myeloblastosis*	<0.001	<0.001	81.26%	73.31%	1.11	IRF3(IRF)	*Interferon Regulatory Family*	0.01	0.131	20.83%	12.54%	1.66
LBD23	*Lateral organs boundary domain*	<0.001	<0.001	49.03%	40.07%	1.22	MYB3R5(MYB)	*Myeloblastosis*	0.01	0.131	5.95%	1.96%	3.04
ZNF519(Zf)	*Zinc-finger protein*	0.01	0.6741	45.45%	8.39%	5.42	Elk4(ETS)	*E twenty six*	0.001	0.242	42.99%	26.90%	1.60
Mef2c(MADS)	*Minichromosome maintenance 1, agamous, deficiens, serum response factor*	0.01	1	45.45%	12.37%	3.67	ZBTB18(Zf)	*Zinc-finger protein*	0.01	0.632	35.51%	22.29%	1.59
TF motif enrichment in hypermethylated genes TF motif enrichment in hypermethylated genes							EHF(ETS)	*E twenty six*	0.01	0.632	63.55%	48.63%	1.31
							ATHB33(ZFHD)	*zinc finger homo domain*	0.01	0.632	47.66%	33.29%	1.43
							OCT4-SOX2-TCF-NANOG	*Stem cell pluripotency regulatory factors*	0.01	0.632	12.15%	4.69%	2.59
							Tcf12(bHLH)	*Basic helix loop helix*	0.01	0.632	56.07%	42.13%	1.33
							AT2G15740(C2H2)	*C2H2 zinc finger domain*	0.01	0.632	31.78%	20.22%	1.57
							EWS:FLI1-fusion(ETS)	*E twenty six*	0.01	0.632	36.45%	24.42%	1.49
							IRF3(IRF)	*Interferon Regulatory Family*	0.01	0.632	19.63%	10.55%	1.86
							IRF1(IRF)	*Interferon Regulatory Family*	0.01	0.632	11.21%	4.59%	2.44
							ISRE(IRF)	*Interferon Regulatory Family*	0.01	0.632	6.54%	1.87%	3.50
							Mef2b(MADS)	*Minichromosome maintenance 1, agamous, deficiens, serum response factor*	0.01	0.632	34.58%	23.01%	1.50
							Ascl2(bHLH)	*Basic helix loop helix*	0.01	0.632	63.55%	50.63%	1.26
							ETS:RUNX (ETS, Runt)	*E twenty six*	0.01	0.632	10.28%	4.14%	2.48
							HEB(bHLH)	*Basic helix loop helix*	0.01	0.632	82.24%	70.92%	1.16

## Data Availability

The raw data supporting the conclusion of this article will be made available by the authors, without undue reservation.

## Supplementary Materials

The following supporting information can be downloaded at: https://www.mdpi.com/article/10.3390/biology14010052/s1. Figure S1: PCA and Distribution of DMRs in Old (18mo) vs Young (3mo) Vehicle Male and Female Mice; Table S1: Other pathways with significant DMRs in male mice that have been reported to be affected by Centella asiatica in the literature [31]; Table S2: Other pathways with significant DMRs in female mice that have been reported to be affected by Centella asiatica in the literature; Table S3: Distribution of methylation in DMRs of old (18mo) and young (3mo) Vehicle mice; Table S4: Top 15 enriched GO biological processes found anywhere in the genome of old (18mo) vs Young (3mo) Vehicle Mice; Table S5: Top 10 enriched mammalian transcription factor binding motifs in hypomethylated genes in the blood of old (18mo) vs young (3mo) vehicle-mice; Table S6: Top 10 enriched mammalian transcription factor binding motifs in Hypermethylated Regions in the blood of old (18mo) vs young (3mo) vehicle-mice.

## Abbreviations

The following abbreviations are used in this manuscript:

DNA	Deoxyribonucleic Acid
RNA	Ribonucleic Acid
CAW	Centella asiatica water extract
CpG	Cytosine followed by Guanine site
RRBS	Reduced representation bisulfite sequencing
DMR(s)	Differentially methylated region(s)
GO	Gene Ontology
PCR	Polymerase chain reaction
EDTA	Ethylenediaminetetraacetic acid
KCVI	Knight Cardiovascular Institute
MSSPR	Massively Parallel Sequencing Shared Resource
NEB	New England Biolabs
HOMER	Hypergeometric Optimization of Motif Enrichment
OHSU	Oregon Health & Science University
NIH	National Institute of Health
TSS	Transcription start site
AT	Activating transcription factor
bHLH	Basic helix loop helix
BZR	Brassinazole-resistant
C2H2	C2H2 zinc-finger domain
CAMTA	Calmodulin-binding transcription activator
CTF	CCAAT box-binding transcription factor
DPL	Doppel Protein(s)
ETS	E-twenty-six
IRF	Interferon Regulatory Family
KLF	Kruppel-like Factors
LBD	Lateral organs boundary domain
MADS	Minichromosome maintenance 1, agamous, deficiens, serum Response factor
MYB	Myeloblastosis
SP	Specialty Protein
Zf	Zinc-finger protein
ZFHD	Zinc-finger homo domain
