# Supplementary material for: Peripheral Blood DNA Methylation Changes in Response to Centella asiatica Treatment in Aged Mice
Source: Biology (Basel). 2025 Jan 10;14(1):52. doi: 10.3390/biology14010052 (PMC11762129; doi:10.3390/biology14010052)
Supplement: Supplementary file 1 [file biology-14-00052-s001.zip › biology-3410573-supplementary.pdf]

Supplemental Material

Supplementary Table S1| Other pathways with significant DMRs in male mice that have been reported to be affected by Centella asiatica in the literature [31]

Regulation of dendritic spine development - Males

	meth diff	p value	q value	location
CAMK2B	-11.701	0.005	0.029	Intron
PTPRS	-10.729	0.001	0.011	intron
DLG5	-10.073	<0.001	0.002	Promoter (<=1kb)
FOXO6	-12.785	<0.001	0.003	Distal Intergenic
KIF1A	-13.139	0.017	0.064	Promoter (<=1kb)
SHANK3	-10.881	<0.001	0.001	Promoter (<=1kb)
SHANK1	-11.846	<0.001	0.003	Promoter (<=1kb)

Calcium ion regulated exocytosis - Males

	meth diff	p value	q value	location
Rims1	-10.707	0.003	0.019	Promoter (<=1kb)
Rab3a	-11.149	0.010	0.045	Promoter (<=1kb)
Syt2	-15.525	0.001	0.008	Promoter (<=1kb)
Syt1	-11.225	0.011	0.048	Promoter (<=1kb)
Syt11	-10.195	<0.001	0.002	Promoter (<=1kb)
Sdf4	-10.286	0.031	0.095	Promoter (<=1kb)
Syt6	-10.558	<0.001	0.002	Promoter (<=1kb)

Calcium ion regulated exocytosis of neurotransmitters - Males

	meth diff	p value	q value	location
Unc13b	-12.478	<0.001	0.006	Promoter (<=1kb)
Rims1	-10.707	0.003	0.019	Promoter (<=1kb)
Syt2	-15.525	0.001	0.008	Promoter (<=1kb)
Syt1	-11.225	0.011	0.048	Promoter (<=1kb)
Syt11	-10.195	<0.001	0.002	Promoter (<=1kb)

Supplementary Table S2| Other pathways with significant DMRs in female mice that have been reported to be affected by Centella asiatica in the literature

Regulation of dendritic cell apoptotic process - Females

	meth diff	p value	q value	location
Rapgef2	10.176	0.003	0.065	Promoter (<=1kb)
Lgals9	12.168	0.002	0.059	Distal Intergenic

Nerve Growth Factor signaling pathway- Females

	meth diff	p value	q value	location
Rapgef1	-23.373	0.002	0.063	Intron
Rapgef2	10.176	0.003	0.065	Promoter (<=1kb)

CAMP biosynthetic Process - Females

	meth diff	p value	q value	location
Adcy2	11.188	0.003	0.070	Promoter (<=1kb)
Adcy7	13.319	0.000	0.008	Intron

Supplemental Table S3| Distribution of methylation in DMRs of old (18mo) and young (3mo) Vehicle mice

Comparison (A vs. B)	Total Number Sig DMRs	Number of Significant Hypomethylated DMRs	Number of Significant Hypermethylated DMRs
Male old vs young	338	166	172
Female old vs young	197	168	29

Supplemental Figure S1| PCA and Distribution of DMRs in Old (18mo) vs Young (3mo) Vehicle Male and Female Mice

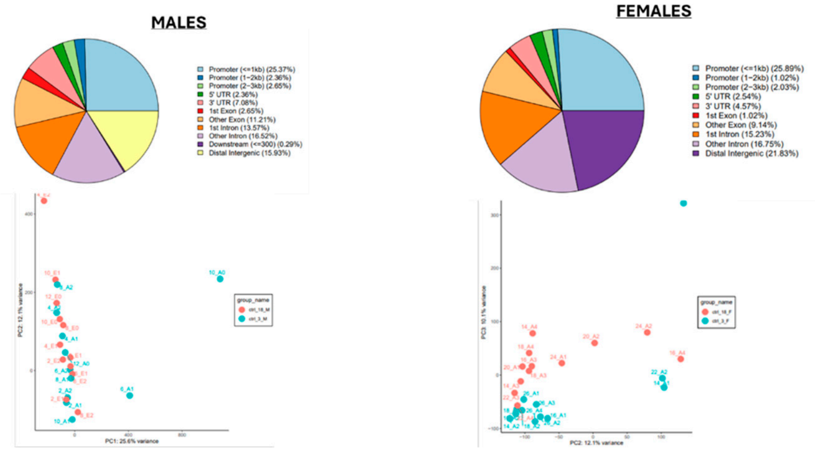

Supplemental Table S4| Top 15 enriched GO biological processes found anywhere in the genome of old *(18mo) vs* Young *(3mo) Vehicle Mice*

MALES

Endoderm Formation (GO:0001706)	0.00001416	0.01969
Positive Regulation Of Cell Projection Organization	0.00008926	0.06208
Regulation Of Neuron Projection Development (GO:0010975)	0.0003786	0.1387
Regulation Of Microtubule-Based Process (GO:0032886)	0.0003989	0.1387
Positive Regulation Of Neuron Projection Development	0.0005496	0.1426
Semaphorin-Plexin Signaling Pathway Involved In Neuron Projection Guidance (GO:1902285)	0.0007177	0.1426
Positive Regulation Of Regulated Secretory Pathway	0.0007177	0.1426
Regulation Of Calcium Ion-Dependent Exocytosis	0.0008435	0.1467
Chondrocyte Differentiation (GO:0002062)	0.0009667	0.1494
Endodermal Cell Differentiation (GO:0035987)	0.00125	0.1739
Semaphorin-Plexin Signaling Pathway (GO:0071526)	0.001777	0.2247
Regulation Of Cardiac Muscle Contraction (GO:0055117)	0.001982	0.2298
Positive Regulation Of Lipoprotein Particle Clearance	0.002292	0.2453
Regulation Of Ventricular Cardiac Muscle Cell Membrane Repolarization (GO:0060307)	0.002486	0.247
Positive Regulation Of Transcription By RNA Polymerase II	0.00287	0.2481

FEMALES

Cellular Response To Oxygen-Containing Compound	0.000008261	0.01121
Regulation Of miRNA Transcription (GO:1902893)	0.00002014	0.01367
Cellular Response To Retinoic Acid (GO:0071300)	0.00004167	0.01885
Actin Cytoskeleton Reorganization (GO:0031532)	0.0001455	0.04937
Response To Retinoic Acid (GO:0032526)	0.0002625	0.07124
Regulation Of Blood Vessel Endothelial Cell Proliferation Involved In Sprouting Angiogenesis (GO:1903587)	0.0004314	0.09388
Non-Canonical Wnt Signaling Pathway (GO:0035567)	0.0006071	0.09388
Positive Regulation Of miRNA Transcription (GO:1902895)	0.000729	0.09388
Response To Ketone (GO:1901654)	0.000854	0.09388
Regulation Of Long-Chain Fatty Acid Import Across Plasma Membrane (GO:0010746)	0.000881	0.09388
Regulation Of cGMP-mediated Signaling (GO:0010752)	0.000881	0.09388
Regulation Of Protein Localization To Plasma Membrane	0.0009917	0.09388
Positive Regulation Of Smooth Muscle Cell Proliferation	0.001023	0.09388
Positive Regulation Of miRNA Metabolic Process	0.001197	0.09388
Positive Regulation Of Endothelial Cell Migration	0.001303	0.09388

Supplemental Table S5| Top 10 enriched mammalian transcription factor binding motifs in hypomethylated genes in the blood of old (18mo) vs young (3mo) vehicle-mice.

MALES

		P value	q value	% of Targets Sequences	% of Background Sequences	% over background
1	Elk4(ETS)	1.00E-10	0	45.18%	21.66%	23.52%
2	Elk1(ETS)	1.00E-07	0	40.96%	22.37%	18.59%
3	Fli1(ETS)	1.00E-06	0.0001	62.65%	42.77%	19.88%
4	ELF1(ETS)	1.00E-06	0.0001	37.95%	20.65%	17.30%
5	ERG(ETS)	1.00E-05	0.0003	75.90%	58.41%	17.49%
6	GABPA(ETS)	1.00E-05	0.0003	54.22%	36.31%	17.91%
7	ETV4(ETS)	1.00E-05	0.0004	62.05%	44.23%	17.82%
8	EWS:FLI1-fusion(ETS)	1.00E-04	0.0017	39.16%	24.28%	14.88%
9	ETS(ETS)	1.00E-04	0.0068	24.70%	13.42%	11.28%
10	IRF2(IRF)	1.00E-04	0.0068	11.45%	4.15%	7.30%

FEMALES

		P value	q value	% of Targets Sequences	% of Background Sequences	% over background
1	Elk4(ETS)	1.00E-07	0.0001	42.86%	24.00%	18.86%
2	NF1(CTF)	1.00E-06	0.0002	33.33%	17.25%	16.08%
3	ETV4(ETS)	1.00E-05	0.0008	64.29%	46.45%	17.84%
4	Elk1(ETS)	1.00E-05	0.0009	41.07%	25.01%	16.06%
5	AT5G05550(Trihelix)	1.00E-05	0.0014	61.90%	44.91%	16.99%
6	Fli1(ETS)	1.00E-04	0.0069	60.12%	44.67%	15.45%
7	Unknown3	1.00E-04	0.0069	14.29%	5.82%	8.47%
8	ERG(ETS)	1.00E-03	0.0192	72.02%	58.28%	13.74%
9	IRF8(IRF)	1.00E-03	0.0362	22.62%	12.82%	9.80%
10	At4g18890(BZR)	1.00E-03	0.0561	14.29%	6.89%	7.40%

Supplemental Table S6| Top 10 enriched mammalian transcription factor binding motifs in Hypermethylated Regions in the blood of old (18mo) vs young (3mo) vehicle-mice.

MALES

		P value	q value	% of Targets Sequences	% of Background Sequences	% over background
1	HIF2a(bHLH)	1.00E-04	0.0303	28.32%	15.99%	12.33%
2	COUP-TFII(NR)	1.00E-03	0.1003	72.25%	59.72%	12.53%
3	SPDEF(ETS)	1.00E-03	0.1003	53.18%	40.60%	12.58%
4	GATA14(C2C2gata)	1.00E-03	0.1003	35.26%	24.04%	11.22%
5	LXRE(NR)	1.00E-03	0.1003	6.36%	1.93%	4.43%
6	Sox4(HMG)	1.00E-02	0.1413	38.73%	27.95%	10.78%
7	RIN(MADS)	1.00E-02	0.1413	42.20%	31.22%	10.98%
8	NFE2L2(bZIP)	1.00E-02	0.1413	4.62%	1.24%	3.38%
9	ANAC103(NAC)	1.00E-02	0.1413	20.23%	12.10%	8.13%
10	ELF1(ETS)	1.00E-02	0.1413	39.31%	28.71%	10.60%

FEMALES

		P value	q value	% of Targets Sequences	% of Background Sequences	% over background
1	CDF3(C2C2dof)	1.00E-02	1	79.31%	52.77%	26.54%
2	COUP-TFII(NR)	1.00E-02	1	86.21%	62.55%	23.66%
3	ETV4(ETS)	1.00E-02	1	72.41%	48.47%	23.94%
4	EAR2(NR)	1.00E-02	1	75.86%	52.73%	23.13%
5	IRF4(IRF)	1.00E-02	1	34.48%	15.49%	18.99%
